# QRS complex and T wave planarity for the efficacy prediction of automatic implantable defibrillators

**DOI:** 10.1136/heartjnl-2023-322878

**Published:** 2023-09-15

**Authors:** Katerina Hnatkova, Irena Andršová, Tomáš Novotný, Bert Vanderberk, David Sprenkeler, Juhani Junttila, Tobias Reichlin, Simon Schlögl, Marc A Vos, Tim Friede, Axel Bauer, Heikki V Huikuri, Rik Willems, Georg Schmidt, Christian Sticherling, Markus Zabel, Marek Malik

**Affiliations:** 1 National Heart and Lung Institute, Imperial College London, London, UK; 2 Department of Internal Medicine and Cardiology, University Hospital Brno, Brno, Czech Republic; 3 Department of Internal Medicine and Cardiology, Masaryk University, Brno, Czech Republic; 4 Department of Cardiovascular Sciences, University of Leuven, Leuven, Belgium; 5 Department of Medical Physiology, University Medical Center Utrecht, Utrecht, The Netherlands; 6 MRC Oulu, University Central Hospital of Oulu and University of Oulu, Oulu, Finland; 7 Department of Cardiology, Inselspital, Bern University Hospital, Bern, Switzerland; 8 Department of Cardiology and Pneumology, University Medical Center Göttingen, Gottingen, Germany; 9 German Center of Cardiovascular Research (DZHK), partner site Göttingen, Göttingen, Germany; 10 Department of Medical Statistics, University Medical Center Göttingen, Göttingen, Germany; 11 University Hospital for Internal Medicine III, Medical University Innsbruck, Innsbruck, Austria; 12 University Central Hospital of Oulu and University of Oulu, Oulu, Finland; 13 Division of Experimental Cardiology, Department of Cardiovascular Diseases, University of Leuven, Leuven, Belgium; 14 Division of Clinical Cardiology, University Hospitals Leuven, Leuven, Belgium; 15 Medizinische Klinik, Klinikum rechts der Isar der Technischen Universität München, Munich, Germany; 16 Department of Cardiology, University Hospital of Basel, Basel, Switzerland; 17 Cardiology and Pneumology, Heart Center, University Hospital Göttingen, Göttingen, Germany

**Keywords:** Defibrillators, Implantable, Biomarkers, Electrocardiography

## Abstract

**Objective:**

To test the hypothesis that in recipients of primary prophylactic implantable cardioverter-defibrillators (ICDs), the non-planarity of ECG vector loops predicts (a) deaths despite ICD protection and (b) appropriate ICD shocks.

**Methods:**

Digital pre-implant ECGs were collected in 1948 ICD recipients: 21.4% females, median age 65 years, 61.5% ischaemic heart disease (IHD). QRS and T wave three-dimensional loops were constructed using singular value decomposition that allowed to measure the vector loop planarity. The non-planarity, that is, the twist of the three-dimensional loops out of a single plane, was related to all-cause mortality (n=294; 15.3% females; 68.7% IHD) and appropriate ICD shocks (n=162; 10.5% females; 87.7% IHD) during 5-year follow-up after device implantation. Using multivariable Cox regression, the predictive power of QRS and T wave non-planarity was compared with that of age, heart rate, left ventricular ejection fraction, QRS duration, spatial QRS-T angle, QTc interval and T-peak to T-end interval.

**Results:**

QRS non-planarity was significantly (p<0.001) associated with follow-up deaths despite ICD protection with HR of 1.339 (95% CI 1.165 to 1.540) but was only univariably associated with appropriate ICD shocks. Non-planarity of the T wave loop was the only ECG-derived index significantly (p<0.001) associated with appropriate ICD shocks with multivariable Cox regression HR of 1.364 (1.180 to 1.576) but was not associated with follow-up mortality.

**Conclusions:**

The analysed data suggest that QRS and T wave non-planarity might offer distinction between patients who are at greater risk of death despite ICD protection and those who are likely to use the defibrillator protection.

**WHAT IS ALREADY KNOWN ON THIS TOPIC**

- In normal physiological recordings, the vectorcardiographic loops of the QRS complex and of the T wave are known to be practically planar, that is, with no or only little three-dimensional twist. The non-planarity of the vectorcardiographic loops has previously been observed mainly in patients with ischaemic heart disease. Nevertheless, the extent of the twist of these loops has not been investigated in recipients of implantable cardioverter-defibrillators (ICDs) implanted for primary prophylactic reasons.

**WHAT THIS STUDY ADDS**

- The spatial twist of the vectorcardiographic loops of the QRS complex and of the T wave is measurable not only based on standard 12-lead ECGs but also based on restricted electrode sets suitable for prolonged monitoring.
- In primary prophylactic ICD recipients, the extent of the twist of the QRS complex and of the T wave was found to be an independent predictor of risk.
- The twist of the QRS complex was found to predict both all-cause mortality and, to a lesser extent, appropriate ICD shocks.
- The twist of the T wave strongly predicted appropriate ICD shocks.

**HOW THIS STUDY MIGHT AFFECT RESEARCH, PRACTICE OR POLICY**

- The results of the study need to be replicated in independent datasets. If the results are confirmed in prospectively collected ECGs of primary prophylactic ICD recipients, the predictive value of the QRS and T wave spatial twists needs to be assessed in terms of the distinction between patients who do and do not benefit from ICD primary prophylaxis. Such investigations might eventually lead to a change in the selection of patients for ICD primary prophylaxis.

## Introduction

QRS micro-fragmentation was recently proposed to characterise depolarisation abnormalities beyond the visual detection on standard 12-lead ECGs.^1^ This characteristic was shown to provide an independent mortality predictor in different populations.^2^ It was proposed that the QRS micro-fragmentation expresses localised irregularities of ventricular excitation and that these aberrations signify heart failure including the early subclinical stages.

The essential concept of micro-fragmentation assessment is based on the analysis of simultaneously recorded ECG leads (ie, of the eight mutually independent signals of the standard ECG recording). The use of standard clinical ECGs makes the QRS micro-fragmentation analysis widely applicable to different clinical settings. Nevertheless, reliance on multiple ECG leads makes it inapplicable to situations when fewer ECG leads are available, for example, monitoring systems, standard clinical Holters and wearable ECG devices.

It has previously been reported that in physiological ECG recordings, the vectorcardiographic (VCG) loops of the QRS complex are essentially planarly,^3^ that is, that the three-dimensional QRS dipole moves practically in a single plane the orientation of which depends on the position of the organ. Similar observations were also made for the VCG loop of the T wave.^4^

Our understanding of the predictive power of QRS micro-fragmentation suggests that aberrations of the depolarisation sequence might also influence the three-dimensional QRS loop and twist it outside a single two-dimensional plane. Since the VCG loops might be, in principle, constructed from as few as three independent ECG leads, the non-planarity characteristics might be derived from limited electrode sets.

Being guided by these considerations, we tested the hypotheses that QRS complex and T wave non-planarity indices are factors predicting death despite defibrillator protection and appropriate shock therapy by implantable cardioverter-defibrillators (ICDs). These hypotheses were tested in the previously reported population of recipients of prophylactic ICDs collected within the retrospective part of the EU-CERT-ICD Study.^5^

## Methods

### Population and follow-up data

As already published,^2 5^ the EU-CERT-ICD Study included a retrospective part that collected data of ICD recipients implanted in different European centres for primary prophylactic reasons between 2000 and 2014. Baseline characteristics, including age at ICD implantation, pre-implantation left ventricular ejection fraction (LVEF), and the distinction between ischaemic and non-ischaemic heart disease, were collected at each centre. Follow-up data were also provided by participating centres and were quality controlled by the team of University Hospital of Basel, Switzerland.

In each case, the ICD programming corresponded to the clinical needs and to the standard practice of each centre. The follow-up data provided by individual centres included all-cause mortality and ICD shocks that were adjudicated to differentiate between appropriate and inappropriate shocks. For the purposes of this investigation, all-cause mortality and appropriate ICD shocks were used as two separate follow-up event categories. Time of survival was defined as the interval between the ICD implantation and death; patients who did not die were censored at the end of the follow-up by the relevant centre. For patients who experienced an appropriate ICD shock, the interval between the device implantation and the first such shock was considered. As with all-cause mortality, patients who did not experience an appropriate ICD shock were censored (for the purposes of ICD shock prediction) at the time of their death or at the follow-up end. For the purposes of this investigation, follow-up was restricted to the first 5 years after ICD implantation.

### ECG recordings

Electronic 12-lead short-term ECGs were obtained in 1948 patients prior to ICD implantation (median 1 day before implantation, IQR 1–6 days). These patients constituted the population of this study and were collected and followed up at the Department of Cardiology, University Hospital of Basel, Switzerland (n=488); Department of Cardiology and Pneumology, University Medical Center, Göttingen, Germany (n=441); Department of Cardiovascular Sciences, University of Leuven, Belgium (n=361); University Central Hospital of Oulu, Finland (n=32); and Department of Medical Physiology, University Medical Center Utrecht, the Netherlands (n=626). The ECG recordings obtained at Oulu were of 8-second duration, other ECGs were of 10-second duration.

### ECG measurement

As previously reported,^2^ the signals of all the ECGs were converted to the same digital format, cubic spline resampled (where appropriate) to 1 kHz frequency and filtered. Automatic QRS detection was visually confirmed and used to construct representative median beatforms of each ECG lead. For each ECG, these representative beatforms of different leads were superimposed on the same isoelectric axis and previously described algorithms were used to detect QRS onset, QRS offset and T wave offset. In each ECG, the delineation positions were visually checked and, where appropriate, manually corrected using a computer display with a single millisecond precision.

Using these representative beatform delineations, QRS complex and QT interval durations were obtained. Heart rate was derived from the average of all RR intervals in the entire ECG. Using this heart rate, rate-corrected QTc intervals were derived by Fridericia formula. The T-peak to T-end (TpTe) intervals were measured in the vector magnitude of all 12 leads using a previously reported method.^6^ Using also previously published technique,^4^ spatial QRS-T angles were measured and expressed in degrees (between 0° and 180°).

### Assessment of QRS and T wave planarity

**Figure 1:**
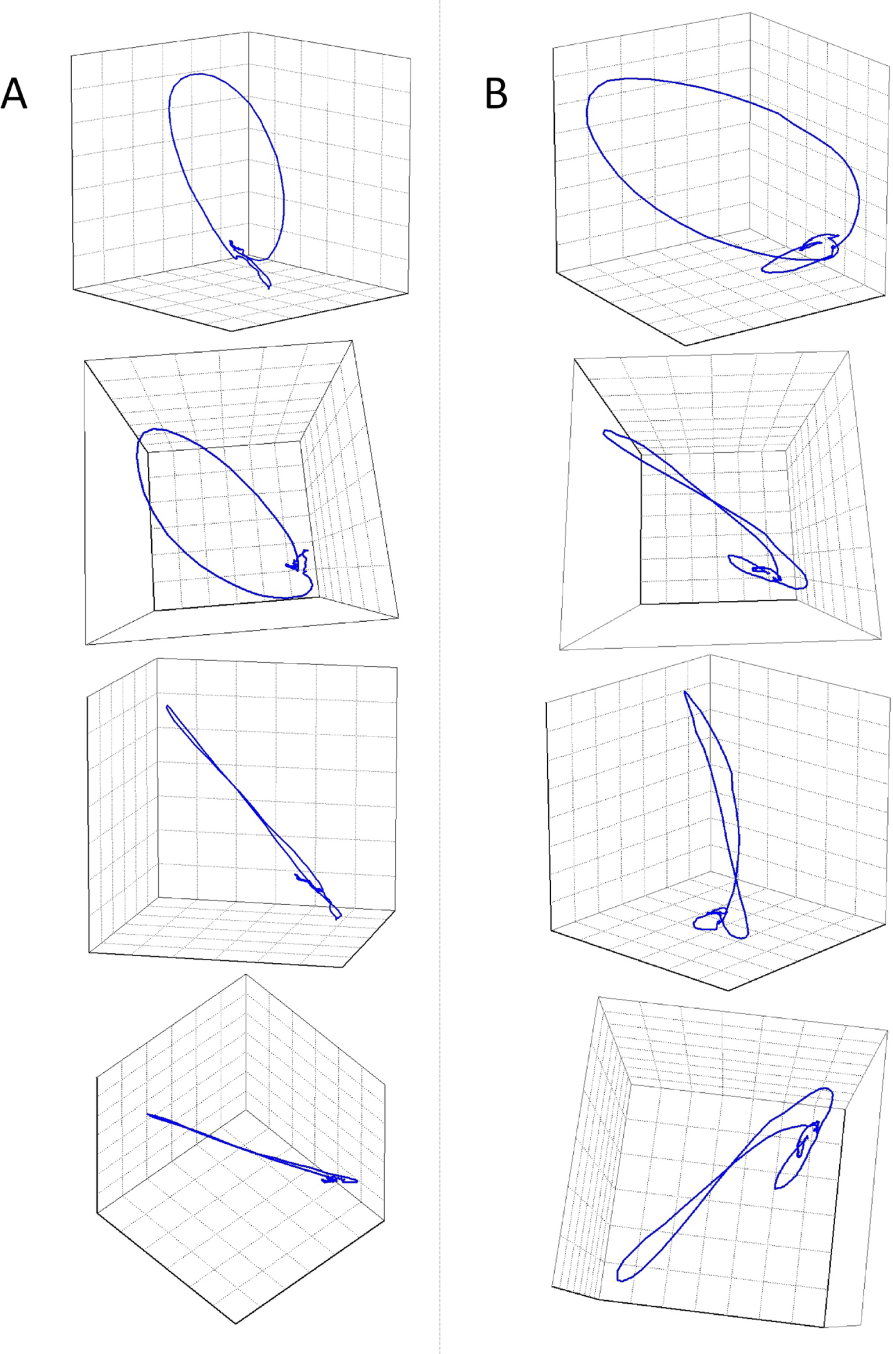
Examples of QRS planarity assessment. The images in each of the two columns of figure show different projections of three-dimensional QRS complex loops of ECGs of two different study subjects (ie, each column of the figure shows different views on the very same QRS complex loop), both around 70 years of age, with similar underlying heart rate and with the same QRS duration (see online supplemental material for the images of source ECGs). The images in (A) show that the loop of the QRS complex was planar, that is, that it collapses into a practically straight line when viewed from the side of the plane of the QRS vector movement. The QRS non-planarity (ie, the departures from the plane of the vector movement) was 2.17%; the patient survived the study follow-up. On the contrary, the images in (B) show that the loop of the QRS complex vector movement was twisted out of a single plane and that consequently, its three-dimensional nature was visible in all possible projections. The QRS non-planarity was 10.9% and the patient died 278 days after ICD implantation. The T wave planarity was assessed in the same way (note that small T wave loops are also visible in the images). Online supplemental material explains the techniques of the non-planarity measurements. Supplemental animations of the two QRS loops presented in the figure display the planarity and non-planarity differences of these two cases more clearly. The axes in the animations are the same as in the panels of this figure—in both ECGs; the axes were derived by singular value decomposition and subsequently rotated for display purposes. ICD, implantable cardioverter-defibrillator.

The main plane of ECG vector movement was defined by the means of singular value decomposition (SVD) of the ECG signals. In the same way as previously used for the QRS micro-fragmentation expression, the SVD decomposing signals were obtained.^1^ The first two components (see the online supplemental material for details) defined the main plane and the two-dimensional vector loop movement within this plane. The third component (orthogonal to the first two) expressed the contribution of the signal components that twisted the vector loop outside the main plane (figure 1). The relative extent (percentage) of the third component expressed the non-planarity (the twist) of the three-dimensional vector loop (figure 1).

10.1136/heartjnl-2023-322878.supp1 Supplementary data

Using this principle, two pairs of assessments of the planarity ECG components were computed. First, all algebraically independent leads of the source ECG (that is, leads I, II and V1–V6) were used to apply the SVD algorithm to the QRS complex (the signal between the verified QRS onset and offset) and to the T wave (the signal between the QRS offset and T wave offset).

Second, to model the situation of ECGs with restricted leads, only signals between electrodes V1 and V6 were considered. Potential differences between four electrodes defined three leads, namely V2-V1, V5-V2 and V6-V5 approximating signal acquisition by a chest belt. These three derived leads were subsequently processed by SVD and the same approach as described expressed the QRS and T wave planarity values.

### Statistics and data presentation

Continuous data are presented as medians (IQR). Non-parametric Kruskal-Wallis, Kolmogorov-Smirnov and χ^2^ tests were used for group comparisons of continuous and categorical data, respectively. Non-parametric Spearman correlation coefficients were used to assess and test pairwise associations between continuous variables. Association of variables with outcome variables was tested by Cox regression analysis which was used both with single variables and for multivariable modelling with backwards stepwise elimination. For the purposes of Cox modelling of continuous variables, the QRS complex and T wave non-planarity values were logarithmically transformed. In addition to Cox regression analysis using continuous variables, models with dichotomised variables were used. For this purpose, age was dichotomised at 75 years, heart rate at 75 beats/min, LVEF at 25% (close to the population median), QRS duration at 120 ms^7^, QTc interval at 450 ms^8^, TpTe interval at 100 ms^9^ and QRS-T angle at 110°.^10^ The QRS and T wave non-planarity values were dichotomised at their population medians. Two different datasets were used for multivariable Cox regression analysis. Model 1 tested available variables against the QRS complex and T wave non-planarity values derived from all original eight independent ECG leads; model 2 used QRS complex and planarity values derived from the modelled chest belt of electrodes V1, V2, V5 and V6. The association of the dichotomised non-planarity values with outcome was also tested by Kaplan-Meier survival curves; the differences between the curves were tested by log rank test.

SVD computation and the assessment of non-planarity of QRS complex and of T wave were programmed in C++ (Microsoft Visual Studio Professional 2022, 64-bit V.17.3.5). Statistical evaluation was performed by SPSS package (V.27; IBM Corporation); p values below 0.05 were considered statistically significant.

Additional analyses are described in the online supplemental material.

## Results

**Figure 2:**
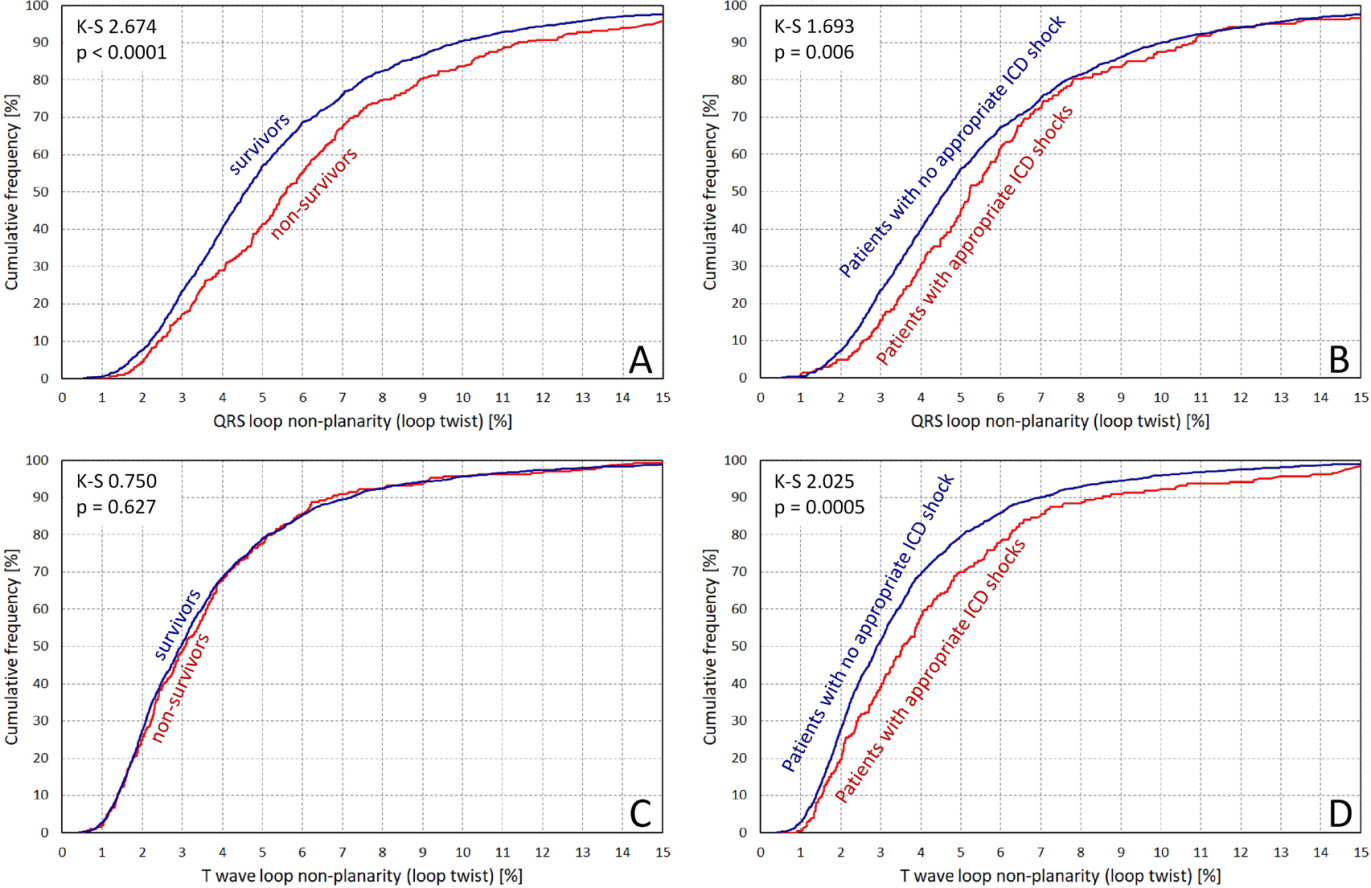
Comparison of QRS complex non-planarity values (A,B) and T wave non-planarity values (C,D) in patients who did and did not survive during the study follow-up (A and C) and in patients who experienced and did not experience appropriate ICD shocks (B and D). In each panel, cumulative distributions of the non-planarity values are shown together with Kolmogorov-Smirnov statistics and their corresponding p values. The non-planarity values shown were derived from the analysis of all eight independent leads of standard 12-lead ECGs. See the online supplemental material for the same comparison of non-planarity values derived from the ECG leads of the modelled chest belt (see the text for details). ICD, implantable cardioverter-defibrillator.

Of the 1948 patients of the study, 294 (15.1%) died during the follow-up restricted to the first 5 years. Defibrillators with cardiac resynchronisation therapy (CRT) function were implanted in 797 patients. In 57 patients (2.9%), the information on ICD shock therapy was not available. Among the remaining 1897 patients, 207 (10.9%) experienced an appropriate ICD shock during the first 5 years of follow-up. Of these, 45 patients (21.7% of patients who received a shock) subsequently died during the first 5 years of follow-up. Clinical characteristics of the population and the measured non-planarity values of ECG components are shown in table 1. Differences between non-planarity values in patients with and without follow-up events are shown in figure 2.

**Table 1:** Clinical characteristics

	No events in 5 years	Death in 5 years	ICD shock in 5 years	P value
N	1492	294	162	
Female sex	319 (21.4%)	45 (15.3%)	17 (10.5%)	0.002
Ischaemic HD	883 (59.2%)	202 (68.7%)	142 (87.7%)	0.003
Non-ischaemic HD	595 (39.9%)	86 (29.3%)	49 (30.2%)	0.007
CRT-D device implanted	589 (39.5%)	159 (54.1%)	73 (45.1%)	<0.001
Age (years)	64.0 (55.0–71.2)	69.3 (62.5–74.8)	63.8 (55.2–70.4)	<0.001
Heart rate (bpm)	69.0 (59.9–79.4)	72.9 (65.1–83.9)	68.0 (58.3–77.5)	<0.001
LVEF (%)	26 (21–31)	25 (20–30)	25 (20–31)	<0.001
QRS (ms)	127 (112–159)	146 (122–171)	131 (114–158)	<0.001
QRS-T angle (^o^)	151.3 (117.0–165.2)	159.9 (142.8–167.4)	148.3 (109.3–162.1)	<0.001
QRS n-pl (8 leads) (%)	4.49 (3.06–6.88)	5.59 (3.55–8.20)	5.21 (3.76–7.05)	<0.001
T wave n-pl (8 leads) (%)	2.87 (1.90–4.51)	3.06 (2.00–4.66)	3.55 (2.36–5.67)	<0.001
QRS n-pl (3 leads) (%)	4.34 (2.83–6.77)	6.00 (3.65–9.08)	4.55 (2.90–6.87)	<0.001
T wave n-pl (3 leads) (%)	3.03 (1.85–4.94)	3.32 (1.95–5.76)	3.85 (2.29–6.08)	0.001
QTc interval (ms)	442.2 (418.6–467.2)	452.4 (428.3–484.8)	442.1 (421.7–467.1)	<0.001
TpTe interval (ms)	97 (85–113)	98 (84–115)	96.5 (86–109)	0.928

The characteristics are shown for population subgroups stratified according to the events during 5-year follow-up (patients who experienced ICD shocks and subsequently died are included among those who died). Note that in 21 patients, the distinction between ischaemic and non-ischaemic HD was unclassified. The values shown are total count (percentage) for categorical variables, and median value (IQR) for continuous variables. The p values show χ^2^ (for categorical variables) and Kruskal-Wallis (for continuous variables) comparisons of the distribution of the three study subgroups.

bpm, beats per minute; CRT-D, cardiac resynchronisation therapy-defibrillator; HD, heart disease; ICD, implantable cardioverter-defibrillator; LVEF, left ventricular ejection fraction; n-pl, non-planarity of the three-dimensional loop; TpTe, T-peak to T-end.

QRS non-planarity values assessed from original 12-lead ECGs and from the modelled chest belt electrodes were significantly correlated (Spearman’s r=0.455, p<0.001). The same was true for the T wave non-planarity (r=0.429, p<0.001). QRS non-planarity values were also correlated with the QRS duration (r=0.204 and 0.189, both p<0.01, for the values derived from the original ECGs and from the chest belt electrodes, respectively). No significant correlations were found between the T wave non-planarity values and the QT interval duration (r=0.012 and r=−0.004, for the two sets of values, respectively). The correlations between the QRS non-planarity and T wave non-planarity were very modest (r=0.154 and r=0.202, respectively).

Table 2 shows the results of the Cox regression analysis based on the continuous variables. The departure from planarity of the QRS complex loop was found to be a strong predictor of all-cause mortality independent of other factors used in the model. While QRS duration and QTc duration were significantly associated with mortality univariably, they were not found significant in a multivariable analysis. Both the departures from QRS planarity assessed in the complete 12-lead ECG and in the precordial band predicted the mortality strongly.

**Table 2:** Event prediction based on continuous variables

	Univariable analysis			Multivariable analysis: model 1 (8 leads)			Multivariable analysis: model 2 (3 leads)		
	Wald	P value	HR (95% CI)	Wald	P value	HR (95% CI)	Wald	P value	HR (95% CI)
**Prediction of 5-year all-cause mortality**									
Age (years)	**48.9**	**<0.001**	**1.043 (1.031 to 1.055**)	37.5	<0.001	1.038 (1.026 to 1.051)	32.0	<0.001	1.035 (1.023 to 1.048)
Heart rate (bpm)	**30.8**	**<0.001**	**1.019 (1.012 to 1.026**)	24.2	<0.001	1.018 (1.011 to 1.025)	19.5	<0.001	1.016 (1.009 to 1.024)
LVEF (%)	**30.1**	**<0.001**	**0.959 (0.945 to 0.973**)	11.4	0.001	0.972 (0.955 to 0.988)	11.7	0.001	0.972 (0.956 to 0.988)
QRS duration (ms)	**26.6**	**<0.001**	**1.009 (1.006 to 1.013**)						
QTc interval (ms)	**20.3**	**<0.001**	**1.006 (1.004 to 1.009**)						
QRS-T angle (^o^)	**26.1**	**<0.001**	**1.010 (1.006 to 1.014**)	8.88	0.003	1.006 (1.002 to 1.010)	8.13	0.004	1.006 (1.002 to 1.010)
TpTe interval (ms)	1.09	0.296	1.002 (0.998 to 1.006)						
log_2_ (QRS n-pl 8 leads)	**17.5**	**<0.001**	**1.329 (1.163 to 1.519**)	16.8	<0.001	1.339 (1.165 to 1.540)			
log_2_ (T wave n-pl 8 leads)	0.87	0.349	1.061 (0.938 to 1.200)						
log_2_ (QRS n-pl 3 leads)	**35.0**	**<0.001**	**1.437 (1.275 to 1.621**)				20.6	<0.001	1.338 (1.180 to 1.517)
log_2_ (T wave n-pl 3 leads)	3.39	0.066	1.109 (0.993 to 1.237)						
**Prediction of 5-year ICD shocks**									
Age (years)	0.43	0.510	1.004 (0.992 to 1.016)						
Heart rate (bpm)	1.73	0.188	0.994 (0.984 to 1.003)						
LVEF (%)	0.01	0.975	1.000 (0.985 to 1.014)						
QRS duration (ms)	0.23	0.879	1.000 (0.995 to 1.004)						
QTc interval (ms)	0.15	0.696	1.001 (0.997 to 1.004)						
QRS-T angle (^o^)	0.91	0.340	0.998 (0.995 to 1.002)						
TpTe interval (ms)	0.50	0.476	0.998 (0.992 to 1.004)						
log_2_ (QRS n-pl 8 leads)	**6.61**	**0.010**	**1.233 (1.051 to 1.447**)						
log_2_ (T wave n-pl 8 leads)	**17.7**	**<0.001**	**1.364 (1.180 to 1.576**)	17.7	<0.001	1.364 (1.180 to 1.576)			
log_2_ (QRS n-pl 3 leads)	1.33	0.249	1.087 (0.943 to 1.253)						
log_2_ (T wave n-pl 3 leads)	**4.96**	**0.026**	**1.159 (1.018 to 1.321**)				4.96	0.026	1.159 (1.018 to 1.321)

Univariable and multivariable (backwards stepwise elimination) Cox regression analyses for the prediction of outcome events during 5-year follow-up (see the text for the distinction of the two multivariable models). Risk factors entered as continuous variables, n-pl indices of QRS and T wave loops entered after logarithmic transformation. Statistically significant results are shown in bold. In addition to the p values, the levels of Wald statistics are also shown. The grey areas indicate the variables excluded in the different multivariable models.

bpm, beats per minute; ICD, implantable cardioverter-defibrillator; LVEF, left ventricular ejection fraction; n-pl, non-planarity; TpTe, T-peak to T-end.

Interestingly, the departure from the planarity of the T wave loop (regardless of whether assessed in the complete ECG or in the precordial band) was the only significant predictor of appropriate ICD shocks that was not eliminated in the multivariable models. QRS planarity assessed from the complete 12-lead ECG was also a univariable predictor of ICD shocks but, in a multivariable analysis, it was eliminated when the T wave loop non-planarity was included.

Consistent results were found with Cox regression models based on dichotomised variables (table 3). When including the distinction between CRT/non-CRT defibrillators, only increased heart rate and QRS non-planarity survived in the multivariable models of mortality prediction.

**Table 3:** Event prediction based on dichotomised and categorical variables

	Univariable analysis			Multivariable analysis: model 1 (8 leads)			Multivariable analysis: model 2 (3 leads)		
	Wald	P value	HR (95% CI)	Wald	P value	HR (95% CI)	Wald	P value	HR (95% CI)
**Prediction of 5-year all-cause mortality**									
Age >75 years	**32.6**	**<0.001**	**2.181 (1.669 to 2.850)**						
Female sex	0.51	0.476	0.891 (0.648 to 1.224)						
Non-ischaemic aetiology	2.32	0.127	0.834 (0.660 to 1.053)						
CRT-D device implanted	**21.0**	**<0.001**	**1.710 (1.360 to 2.152)**						
Heart rate >75 bpm	**19.7**	**<0.001**	**1.685 (1.338 to 2.121)**	8.00	0.005	2.956 (1.392 to 6.274)	8.06	0.005	2.973 (1.401 to 6.305)
LVEF <25%	**14.5**	**<0.001**	**1.563 (1.241 to 1.967)**						
QRS duration >120 ms	**24.9**	**<0.001**	**2.010 (1.528 to 2.643)**						
QTc >450 ms	**8.05**	**0.005**	**1.393 (1.108 to 1.752)**						
QRS-T angle >110°	**14.2**	**<0.001**	**1.554 (1.235 to 1.954)**						
TpTe >100 ms	0.17	0.895	0.985 (0.783 to 1.238)						
QRS n-pl 8 leads >median	**20.4**	**<0.001**	**1.730 (1.364 to 2.193)**	7.19	0.007	3.051 (1.350 to 6.894)			
T wave n-pl 8 leads >median	0.81	0.367	1.111 (0.884 to 1.397)						
QRS n-pl 3 leads >median	**33.2**	**<0.001**	**2.034 (1.598 to 2.589)**				7.78	0.005	3.189 (1.413 to 7.204)
T wave n-pl 3 leads >median	0.95	0.329	1.121 (0.891 to 1.410)						
**Prediction of 5-year ICD shocks**									
Age >75 years	0.20	0.655	0.909 (0.598 to 1.381)						
Female sex	**7.55**	**0.006**	**0.538 (0.346 to 0.837)**	7.48	0.006	0.539 (0.346 to 0.840)	7.08	0.008	0.548 (0.352 to 0.854)
Non-ischaemic aetiology	**3.97**	**0.046**	**0.751 (0.566 to 0.995)**						
CRT-D device implanted	3.29	0.070	0.768 (0.577 to 1.021)						
Heart rate >75 bpm	2.35	0.125	0.790 (0.584 to 1.068)						
LVEF <25%	<0.01	0.989	1.002 (0.755 to 1.330)						
QRS duration >120 ms	1.65	0.199	1.209 (0.905 to 1.614)						
QTc >450 ms	0.24	0.623	0.933 (0.708 to 1.230)						
QRS-T angle >110°	0.70	0.404	0.888 (0.672 to 1.173)						
TpTe >100 ms	**4.33**	**0.037**	**0.743 (0.562 to 0.983)**						
QRS n-pl 8 leads >median	**11.2**	**0.001**	**1.609 (1.217 to 2.128)**	8.93	0.003	1.536 (1.159 to 2.035)			
T wave n-pl 8 leads >median	**12.2**	**<0.001**	**1.647 (1.245 to 2.180)**	9.52	0.002	1.559 (1.176 to 2.067)			
QRS n-pl 3 leads >median	2.76	0.097	1.261 (0.959 to 1.658)						
T wave n-pl 3 leads >median	**5.43**	**0.020**	**1.389 (1.054 to 1.832)**				5.38	0.020	1.389 (1.052 to 1.833)

Univariable and multivariable (backwards stepwise elimination) Cox regression analyses for the prediction of outcome events during 5-year follow-up (see the text for the distinction of the two multivariable models). Risk factors entered as dichotomised variables, n-pl indices of QRS and T wave loops stratified according to population median. Statistically significant results are shown in bold. In addition to the p values, the levels of Wald statistics are also shown. The grey areas indicate the variables excluded in the different multivariable models.

bpm, beats per minute; CRT-D, cardiac resynchronisation therapy-defibrillator; ICD, implantable cardioverter-defibrillator; LVEF, left ventricular ejection fraction; n-pl, non-planarity; TpTe, T-peak to T-end.

As expected, female sex was strongly associated with the absence of ICD shocks which remained in the multivariable models when the absence of planarity of the T wave (assessed in either way) was added to the model. Univariably but not multivariably, non-ischaemic heart disease predicted fewer ICD shocks. Interestingly, the TpTe interval above 100 ms was also found significantly associated with the ICD shocks but, contrary to expectations, it predicted fewer rather than more frequent shocks. It did not remain significant in multivariable models.

**Figure 3:**
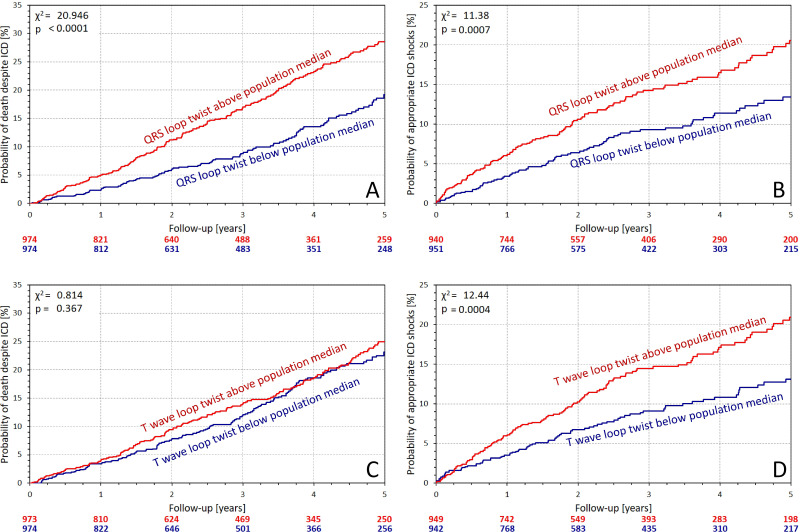
Kaplan-Meier analysis of the probability of death despite ICD protection (A and C) and of the probability of first appropriate ICD shock (B and D). (A,B) Comparison of the subgroups stratified by QRS complex non-planarity (QRS loop twist); (C,D) comparison of the subgroups stratified by the T wave non-planarity (T wave loop twist). The characteristics used in the comparisons were derived from eight independent leads of the complete ECG recordings. χ^2^ statistics and corresponding p values comparing the Kaplan-Meier curves are shown in each panel. The number of patients at risk in these groups is shown below the panels in colours corresponding to the individual graphs. Note that the data on ICD shocks were not available for the complete population—see the text for details. ICD, implantable cardioverter-defibrillator.

**Figure 4:**
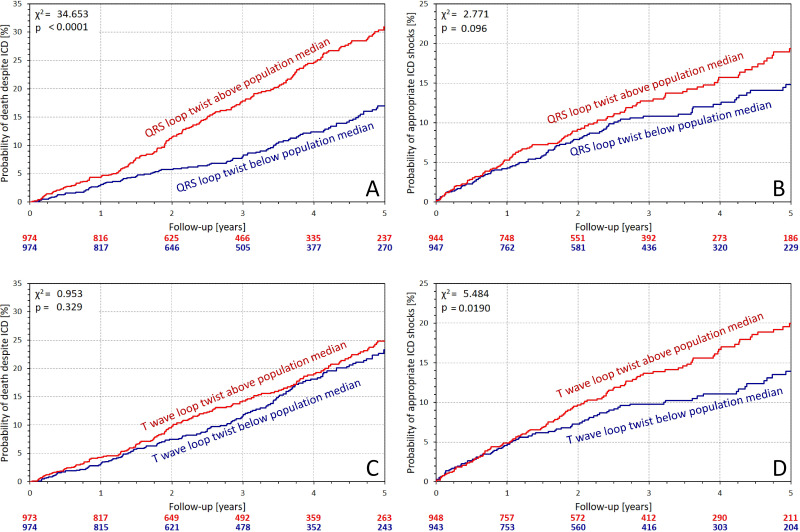
Kaplan-Meier analysis of the probability of death despite ICD protection (A and C) and of the probability of first appropriate ICD shock (B and D). (A,B) Comparison of the subgroups stratified by QRS complex non-planarity (QRS loop twist); (C,D) comparison of the subgroups stratified by the T wave non-planarity (T wave loop twist). The characteristics used in the comparisons were derived from three leads modelling a chest belt (see the text for details). χ^2^ statistics and corresponding p values comparing the Kaplan-Meier curves are shown in each panel. The number of patients at risk in these groups is shown below the panels in colours corresponding to the individual graphs. Note that the data on ICD shocks were not available for the complete population—see the text for details. ICD, implantable cardioverter-defibrillator.

Figures 3 and 4 show the distinction of Kaplan-Meier curves of probability of follow-up events when dividing the population according to QRS complex and T wave loop planarity. All the distinctions were consistent with the univariable results in table 2.

Additional results are described in the online supplemental material.

## Discussion

The analyses show that the non-planarity of the three-dimensional QRS complex and T wave loops provides risk assessment independently of other risk factors.

Physiologically, these observations make sense. Depolarisation abnormalities have repeatedly been associated with poorer survival of cardiac patients^11–13^ and QRS complex abnormalities have been reported to predict heart failure complications more strongly compared with the arrhythmia prediction. Conversely, repolarisation anomalies have repeatedly been associated with arrhythmic complications and sudden cardiac death.^14–16^

The measurement of QRS non-planarity is mathematically independent of the assessment of QRS micro-fragmentation.^1^ We have indeed seen occasional ECGs in which minimal QRS non-planarity was combined with substantial micro-fragmentation and vice versa. Nevertheless, when both QRS non-planarity and QRS micro-fragmentation were used in Cox regression models predicting mortality, the non-planarity was eliminated in the multivariable analysis irrespective of whether continuous or dichotomised variables were used (details not shown). It thus seems that QRS non-planarity, while technically independent, provides less powerful detection of depolarisation abnormalities, although it might be assessed from a limited number of ECG leads. It remains to be seen whether in other populations, QRS non-planarity would significantly contribute to mortality risk predicted by QRS micro-fragmentation.

Since the T wave non-planarity was the only ECG-related parameter that significantly predicted ICD shocks, more advanced analyses of multilead T wave signals of standard ECGs are of interest. Nevertheless, when applying the algorithm of QRS micro-fragmentation to the T wave analysis, we have not obtained any significant prediction of mortality and/or of ICD shocks (details not shown). This is not surprising since propagation of myocardial repolarisation changes is influenced by intercellular electronic interactions^17^ that eliminate abnormalities that would be detectable by the micro-fragmentation analyses. Nevertheless, different analyses of T wave signals might classify pro-arrhythmic abnormalities of the repolarisation sequence more powerfully compared with the simple non-planarity assessment.

Although the measurements of the QRS and T wave non-planarity in full 12-lead ECGs and in the modelled chest belt were significantly correlated, numerical differences were noticeable. This is not surprising. The SVD analysis represents the multilead ECG signal by a multidimensional vector movement which is a simplification of the electrophysiological processes in the complete organ. Irrespective of this simplification, analyses of both electrode configurations led to similar predictions of follow-up events.

The four electrodes that we selected to model a chest belt are not arranged along a true circle which is of advantage when studying three-dimensional ECG signal properties. The three leads that we obtained from these electrodes were selected arbitrarily since any other combinations (eg, V1–V2, V1–V5 and V1–V6) are only algebraic combinations of the used leads and would thus provide the same SVD decomposition results.

Multiple studies investigated the planarity and non-planarity of the QRS complex. The planarity was assessed mainly in VCG using different expressions including the length, width, thickness, and thickness/length and width/length ratios of the QRS loop in different spatial projections.^18^ The QRS non-planarity has previously been investigated mainly in relation to ischaemic heart disease and myocardial infarction.^3 18 19^ Although about 60% of the patients of the study suffered from ischaemic heart disease, multivariable Cox regression analysis showed that the predictive value of QRS loop non-planarity was independent of heart disease aetiology. T wave loop abnormalities have also been repeatedly studied using different characteristics.^4 20^ Among others, T wave-based prediction of mortality in ischaemic heart disease was repeatedly reported.^21–23^ Interestingly, in pilot data that preceded the EU-CERT-ICD Study, Seegers *et al* 24 reported that pre-implantation T wave area predicted appropriate ICD shocks. Of these studies, T wave loop planarity was sparsely investigated, mainly in ischaemic heart disease.^18^

Naturally, every retrospective analysis can only be hypothesis generating. Nevertheless, if our findings are independently confirmed in independent datasets, the assessment of QRS and T loop planarity might be of assistance when considering ICD implantation in patients at the border of criteria of the ICD guidelines. Patients in whom a flat QRS complex loop is combined with a twisted T wave loop might be stronger candidates for ICD prophylaxis compared with patients in whom the planarity of the QRS complex and T wave loops appears to be the other way round. Still, independent confirmations of this possibility are needed before any clinical utility might be proposed.

In future ECG studies, combination of the non-planarity indices of ECG loops needs to be investigated together with other ECG factors^25^ including those that were derived from long-term recordings^26^ which were not available for the retrospective EU-CERT-ICD dataset. Analyses of longer recordings, applicable to both monitoring systems and ECG wearables, would also allow to address the variability and intrasubject reproducibility^27^ of the non-planarity indices.

## Limitations

The available data were limited in several aspects; distinction between cardiac and non-cardiac, and sudden and non-sudden death was not available. While it might be assumed that among prophylactic ICD recipients, mortality was mainly cardiac/cardiovascular, the lack of distinction between sudden and thus presumably arrhythmic and non-sudden deaths prevented us from some more detailed investigations, for example, of the MADIT ICD benefit score.^28^ It might only be assumed that in patients under ICD protection, most cardiac deaths are related to heart failure. CRT utilisation data are not available. Having only one ECG recording in each patient did not allow us to study measurement reproducibility. In the Cox regression analysis, we have intentionally included mostly parameters that might be obtained from ECG analysis. Additional clinical findings, for example, electrophysiological investigation (not available in the investigated dataset), might outperform the risk prediction by the reported indices, although they would not have the advantage of undemanding data acquisition. Finally, these retrospective results have not been tested prospectively. We plan to perform such a prospective evaluation once the follow-up data of the prospective part of the EU-CERT-ICD Study^29^ have been extended; this follow-up extension is presently ongoing.

## Conclusions

Despite these limitations, the study shows that the assessment of QRS complex and T wave loop planarity might also be obtained from limited electrode sets. This makes the assessment plausible also based on data from monitoring systems and wearable devices. In patients with ICD implanted for prophylactic reasons, the estimates of QRS complex and T wave loop planarity appear to differ between those who do and do not use the cardioversion function as well those who do and do not survive despite the ICD anti-tachycardia protection.

10.1136/heartjnl-2023-322878.supp2 Supplementary data

10.1136/heartjnl-2023-322878.supp3 Supplementary data

## Data Availability

Data are available upon reasonable request. Data are available upon reasonable request pending the approval by the EU-CERT-ICD Steering Committee.
