# Supplementary material for: QRS complex and T wave planarity for the efficacy prediction of automatic implantable defibrillators
Source: Heart. 2023 Sep 15;110(3):178–87. doi: 10.1136/heartjnl-2023-322878 (PMC10850677; doi:10.1136/heartjnl-2023-322878)
Supplement: Supplementary data [file heartjnl-2023-322878supp001.pdf]

QRS complex and T wave planarity for the efficacy prediction of automatic implantable defibrillators

by

Katerina Hnatkova[A], Irena Andršová[B,C], Tomáš Novotný[B,C], Bert Vandenberk[D], David J Sprenkeler[E], Juhani Junttila[F], Tobias Reichlin[G], Simon Schlögl[H,I], Marc A Vos[E], Tim Friede[J,I], Axel Bauer[K], Heikki V Huikuri[F], Rik Willems[D], Georg Schmidt[L,M], Christian Sticherling[N], Markus Zabel[H,I], Marek Malik[A,B,C], on behalf of EU-CERT-ICD investigators

[A] National Heart and Lung Institute, Imperial College, London, England,
[B] Department of Internal Medicine and Cardiology, University Hospital Brno, Brno, Czech Republic,
[C] Department of Internal Medicine and Cardiology, Masaryk University, Brno, Czech Republic,
[D] Department of Cardiovascular Sciences, University of Leuven, Leuven, Belgium,
[E] Department of Medical Physiology, University Medical Center Utrecht, Utrecht, The Netherlands,
[F] University Central Hospital of Oulu and University of Oulu, Finland,
[G] Department of Cardiology, Inselspital, Bern University Hospital, Bern, Switzerland
[H] Department of Cardiology and Pneumology, University Medical Center, Göttingen, Germany,
[I] German Center of Cardiovascular Research (DZHK), partner site Göttingen, Göttingen, Germany
[J] Department of Medical Statistics, University Medical Center Göttingen, Göttingen, Germany
[K] University Hospital for Internal Medicine III, Medical University Innsbruck, Innsbruck, Austria,
[L] Klinikum rechts der Isar, Technical University of Munich, Munich, Germany,
[M] German Center for Cardiovascular Research partner site Munich Heart Alliance, Munich, Germany,
[N] Department of Cardiology, University Hospital of Basel, Basel, Switzerland.

Supplementary material

ECG loop planarity measurement

The standard 12-lead ECG contains only 8 algebraically independent leads I, II, V1, V2, …, V6 since the unipolar limb leads are only simple algebraic combinations of leads I and II. Hence, the ECG signal may be considered to constitute a matrix $\mathbb{M}^{8,n}$ of voltage values which has 8 rows $\mathbb{m}_i$ corresponding to individual leads, and n columns, each corresponding to one time-instant. That is, each row $\mathbb{m}_i$ is a function of time and the values $\mathbb{m}_i(t)$, where $0 \leq t < n$, create the image of the i-th lead of the original ECG recording. The singular value decomposition is based on an algorithm that creates a diagonal matrix $\mathbf{\Sigma}^{8,n}$ and matrices $\mathbb{U}^{8,8}$ and $\mathbb{V}^{n,n}$ such that $\mathbf{\Sigma} = \mathbb{U}^T\mathbb{M}\mathbb{V}$, which means $\mathbb{M} = \mathbb{U}\mathbb{W}$, where $\mathbb{W}^{8,n} = \mathbf{\Sigma}\mathbb{V}^T$. Σ is a diagonal matrix with non-zero values only in the left-most diagonal. These elements of $\mathbf{\Sigma}$ (all > 0) are the eigenvalues $\{\sigma_i\}_{i=1}^{8}$ of the decomposition while the columns of matrices $\mathbb{U}$ and $\mathbb{V}$ are the left and right singular vectors. The rows of matrix $\mathbb{W}$ are the algebraically orthogonal components $\{\lambda_i\}_{i=1}^{8}$of the decomposition.

In the signal analyses described in this study, the singular value decomposition was applied to the representative beatforms of each ECG, as described in the main article text.

Original beatform signal in matrix $\mathbb{M}$ can be reconstructed using only a subset of the components of orthogonal signal matrix $\mathbb{W}$. Specifically, for any subset of the 8 orthogonal components (i.e., any selection of one, two, or more of the components λ_i), a matrix $\mathbb{X}^{8,n}$ can be created, for which the rows of the subset are the same as the corresponding rows of $\mathbb{W}$ while other rows contain zeros. The original matrix $\mathbb{M}$ can then be approximated by matrix $\mathbb{N}^{8,n} = \mathbb{U}\mathbb{X}$. Each of the 8 rows of matrix $\mathbb{N}$ correspond to the approximation of the corresponding ECG lead that originally constituted the matrix $\mathbb{M}$.

To quantify the difference between $\mathbb{M}$ and $\mathbb{N}$ signals, this study calculated the area between the signals of individual leads of $\mathbb{M}$ and $\mathbb{N}$. That is for each lead $l \in \{\mathrm{I}, \mathrm{II}, \mathrm{V1}, \mathrm{V2}, \dots, \mathrm{V6}\}$, the difference Δ_l was calculated between original lead $\mathbb{m}_l$ and its approximation $\mathbb{n}_l$ as $\Delta_l = \sum_{t=0}^{n-1}|\mathbb{m}_l(t) - \mathbb{n}_l(t)|$ and the overall difference $\mathbf{\Delta}$ between $\mathbb{M}$ and $\mathbb{N}$ was calculated as the average of all Δ_l (i.e., the average over different algebraically independent leads).

If the selected subset of the 8 orthogonal components was empty, the matrix $\mathbb{N}$ contained only zeros and all $\Delta_l = \sum_{t=0}^{n-1}|\mathbb{m}_l(t)|$.

This allowed to order the orthogonal components $\{\lambda_i\}_{i=1}^{8}$according to their contribution to the original ECG signal. That is, we firstly selected a single component λ_{1st} such that if only this component was used in matrix $\mathbb{X}$, the corresponding $\mathbb{N}$ to $\mathbb{M}$ difference $\mathbf{\Delta}$ was the smallest among all single components λ_i. Subsequently, we selected a second component λ_{2nd} such that if the matrix $\mathbb{X}$

was composed of components ($\lambda_{1st} \oplus \lambda_{2nd}$) – i.e. if the matrix $\mathbb{X}$ had only two non-zero rows, the corresponding $\mathbb{N}$ to $\mathbb{M}$ difference $\mathbf{\Delta}$ was the smallest among all two component combinations ($\lambda_{1st} \oplus \lambda_i$), where $\lambda_i \neq \lambda_{1st}$. The same process was repeated and λ_{3rd} was selected for minimum approximation difference based on ($\lambda_{1st} \oplus \lambda_{2nd} \oplus \lambda_{3rd}$), and so on, up to the selection of the last λ_{8th} orthogonal component. This results in an order of decomposing components $\{\lambda^{(j)}\}_{j=1}^{8}$, where $\lambda^{(1)} = \lambda_{1st}$, $\lambda^{(2)} = \lambda_{2nd}$, and so on.

This selection of λ components results in a sequence of $\mathbb{N}$ to $\mathbb{M}$ differences $\{\mathbf{\Delta}_i\}_{i=0}^{8}$ where $\mathbf{\Delta}_i$ corresponds to the approximation signals $\mathbb{N}$ composed of the first i components selected during the described selection process. Clearly $\mathbf{\Delta}_0 \geq \mathbf{\Delta}_1 \geq \mathbf{\Delta}_2 \geq \cdots \geq \mathbf{\Delta}_8 = 0$. The absolute contribution of the i-th component to the reconstruction of original ECG signal $\mathbb{M}$ is equal to $\mathbf{\Delta}_{i-1} - \mathbf{\Delta}_i$. Clearly, the value of this absolute contribution depends on the magnitude of the original ECG and cannot be directly used for comparisons of different ECGs. For that purpose, it is appropriate to consider the contribution of the i-th component in relative terms, i.e., as a value $\mathbf{\nabla}_i = (\mathbf{\Delta}_{i-1} - \mathbf{\Delta}_i)/\mathbf{\Delta}_0$.

This order of the decomposing components allows to construct a 3-dimensional ECG loop using components $\lambda^{(1)}$, $\lambda^{(2)}$, and $\lambda^{(3)}$. Of these, the components $\lambda^{(1)}$ and $\lambda^{(2)}$ defined the 2-dimensional plane of the vector movement while component $\lambda^{(3)}$ corresponded to the twist of the 3-dimensional loop out of the $\lambda^{(1)}$ + $\lambda^{(2)}$ plane. Correspondingly, the non-planarity of the vector loop was numerically quantified by the value $\mathbf{\nabla}_3$.

When only three independent leads were used modelling the signals between the chest belt electrodes, exactly the same principles were used. The only difference was that the source matrix $\mathbb{M}^{3,n}$ with only 3 rows was considered and thus, only three decomposing components $\{\lambda^{(j)}\}_{j=1}^{3}$ were obtained.

Regardless of whether 8 or only 3 independent ECG leads were used, the values $\mathbf{\nabla}_3$ were obtained using matrices that spanned between the QRS onset and QRS offset, and between the QRS offset and T wave offset.

The examples of 3-dimensional loops shown in Figure 1 of the main article were derived by this signal value decomposition analysis applied to source ECGs shown in Supplementary Figure 1. (Although the source ECGs were of 10-second duration, only 5-second signals are shown in Supplementary Figure 1). The representative beatforms that were processed by the signal value decomposition to create the loops shown in Figure 1 of the main article are presented in Supplementary Figure 2. Note that the differentiation between the QRS loop twists is impossible to judge visually in the Supplementary Figures 1 and 2. See also the supplementary animations.

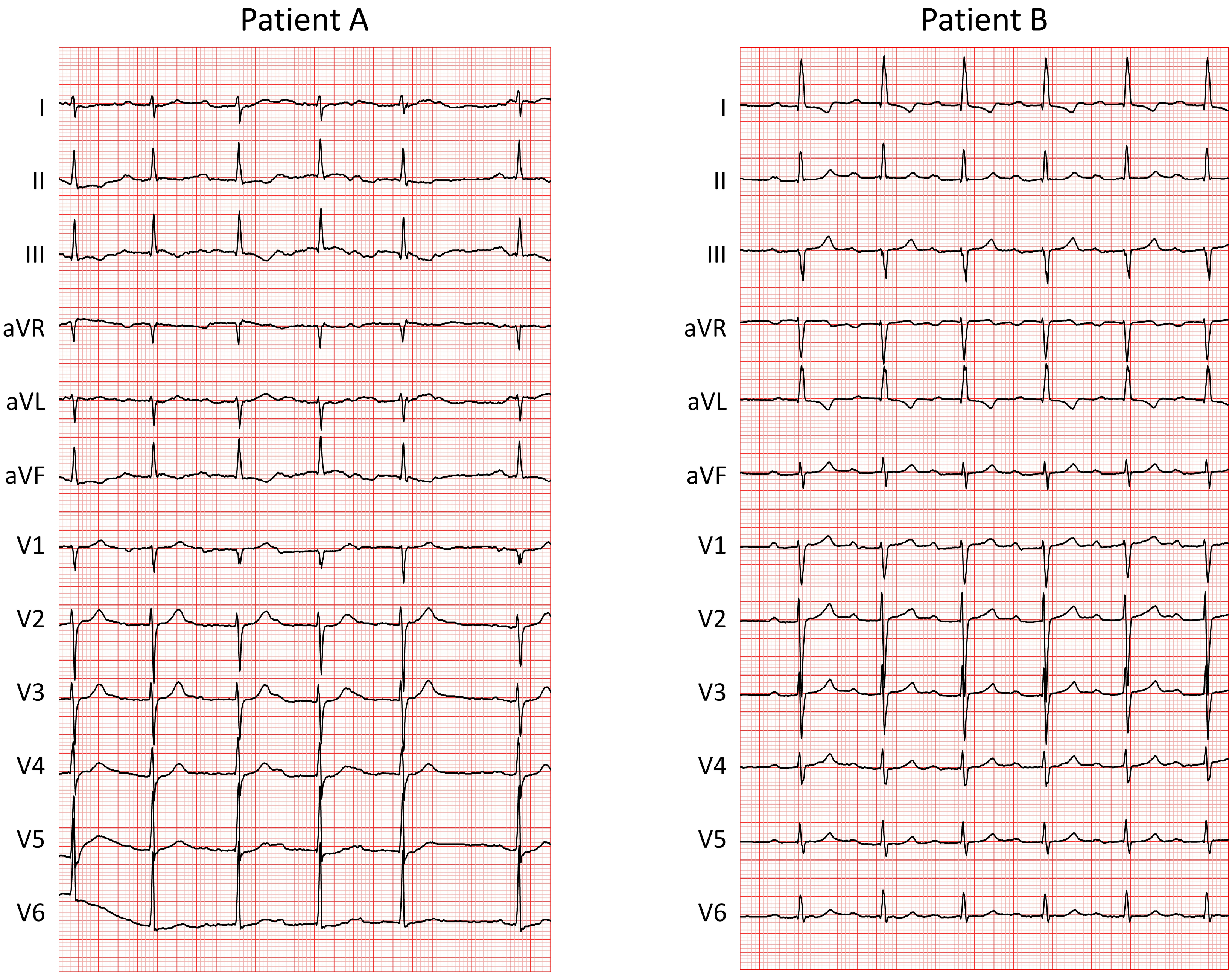

Supplementary Figure 1

BMJ Publishing Group Limited (BMJ) disclaims all liability and responsibility arising from any reliance placed on this supplemental material which has been supplied by the author(s)

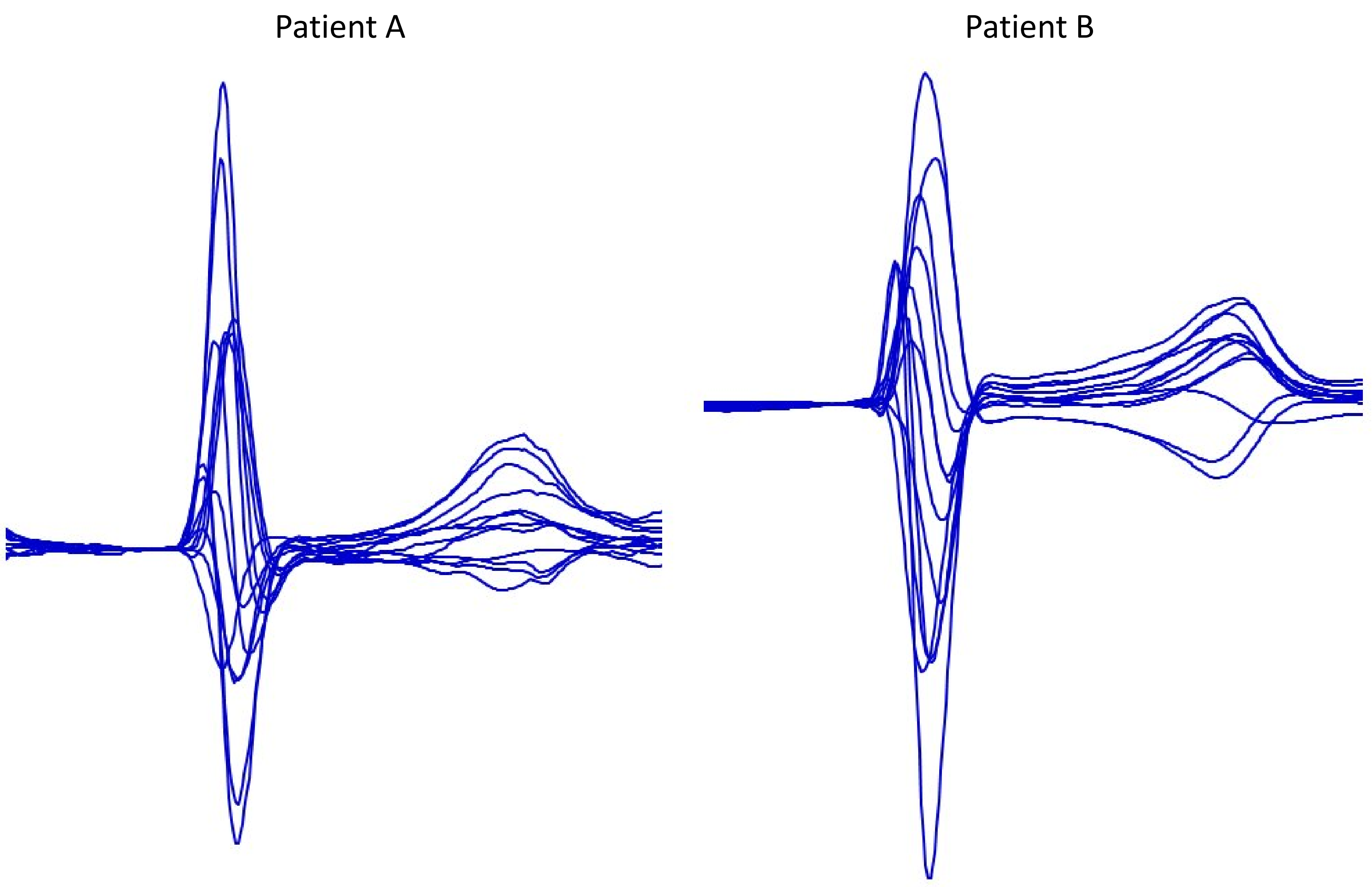

Supplementary Figure 2

Additional data analyses

The comparisons of QRS complex non-planarity values and T wave non-planarity values in patients who did and did not survive during the study follow-up and in patients who experienced and did not experience appropriate ICD shocks, as shown for the values derived from original 12-lead ECGs in Figure 2 of the main article, were repeated for the values derived from the three independent leads modelling the signals between the chest belt electrodes.

As explained in the main article, there were 45 patients who received an appropriate ICD shock but subsequently died before the end of the follow-up. The Kaplan-Meier analyses of the probability of death despite ICD protection, and of the probability of first appropriate ICD shock, as presented in Figures 3 and 4 of the main article, were therefore repeated in populations of patients who did not receive any appropriate ICD shocks during the follow-up (for the comparisons of the probability of death) and who did not die during the follow-up (for the comparisons of the probability of receiving an appropriate ICD shock).

Receiver operator characteristics (i.e., dependencies of specificity on sensitivity) were calculated for the prediction of all-cause mortality and of appropriate ICD shocks before the end of follow-up. These characteristics of the prediction of death were calculated using QRS complex non-planarity data and for the combinations of QRS complex non-planarity with heart rate and the QRS-T angle. The characteristic of the prediction of appropriate ICD shocks was calculated using T wave non-planarity data. The selection of the characteristics used in these calculations was derived from the Cox regression results presented in Table 2 of the main manuscript (while using only ECG-derived data). The characteristics were calculated using the non-planarity measurement derived from the complete 12-lead ECG signals (i.e., from the 8 algebraically independent leads).

The calculation of receiver operator characteristics was performed together with their dual-sided empirical 95% confidence bands. These were calculated using a bootstrap technique with 1000 repetitions. For the calculation of multivariable receiver operator characteristics based on n numerical indices, definition of positive test distinguished between number of m positive comparisons. That is, for the different indices $\{\mathcal{J}_i\}_{i=1}^{n}$, their dichotomies $\{\mathfrak{d}_i\}_{i=1}^{n}$ were varied and for each setting of the dichotomies, the multivariable test was considered positive if $\mathcal{J}_i \geq \mathfrak{d}_i$ for at least m indices. The required number m of positive results was varied between 1 and n.

Applying these general principles to the data of the present study meant that for the combination of heart rate, QRS-T angle, and QRS loop non-planarity, the dichotomies were varied, and the multivariable test was considered positive if 1, or 2, or 3 of these indices were above the

corresponding dichotomy. Since the study date included 294 patients who died during the follow-up, 294 different dichotomies of each of the index had to be considered, leading to 294^3 = 25,412,184 combinations that had to be evaluated to obtain the full sensitivity / specificity profile.

Harrel's concordance index C was computed to further assess discrimination of the risk predictors. This was calculated for QRS-loop non-planarity and T-wave-loop non-planarity used as predictors of all-cause mortality and appropriate ICD shocks. Bootstrap technique with 1000 repetitions was used to obtain confidence intervals of the C index values.

To calibrate the risk predictors, the study population was sorted according to either the QRS-loop non-planarity values or to the T-wave-loop non-planarity values. For both these possibilities, quintiles of the population were considered (numbered 1st with the lowest and 5th with the highest non-planarity values, respectively) and in each quintile, the observed incidence of all-cause mortality and of first appropriate ICD shocks was counted. The non-uniformity of the distribution of these incidence counts was tested using chi-square test.

As explained in the main document, defibrillators with 797 patients (40.9%) were implanted defibrillators with cardiac resynchronisation therapy (CRT) function. The follow-up incidence of all-cause mortality and of appropriate ICD shocks was compared between patients with and without CRT defibrillators. The comparison was based on Kaplan-Meier curves of even probabilities. The comparisons of death probabilities between patients with QRS loop non-planarity above and below population median was also performed separately among patients with and without CRT defibrillators.

Additional results

Supplementary Figure 3 shows the comparisons of QRS complex non-planarity values (panels A and B) and T wave non-planarity values (panels C and D) in patients who did and did not survive during the study follow-up (panels A and C) and in patients who experienced and did not experience appropriate ICD shocks (panels B and D). In each panel, cumulative distributions of the non-planarity values are shown together with Kolmogorov-Smirnov statistics and their corresponding p-values. The non-planarity values shown were derived from the analysis of all 3 independent leads modelling chest belt recordings.

Supplementary Figure 4 shows Kaplan-Meier analyses of the probability of death despite ICD protection (panels A and C) among patients who did not experience any appropriate ICD shocks during study follow-up. The figure further shows Kaplan-Meier analysis of the probability of first appropriate ICD shock (panels B and D) among patients who survived during the study follow-up.

Panels A and B compare the sub-groups stratified by QRS complex non-planarity (QRS loop twist), panels C and D compare the sub-groups stratified by the T wave non-planarity (T wave loop twist). The characteristics used in the comparisons were derived from 8 independent leads of the complete ECG recordings. Chi-square statistics and corresponding p-values comparing the Kaplan-Meier curves are shown in each panel. The number of patients at risk in these groups are shown below the panels in colours corresponding to the individual graphs. Supplementary Figure 5 shows the same Kaplan-Meier analyses as presented in Supplementary Figure 4 but instead of characteristics derived from 8 independent leads of the complete ECG recordings, it shows the comparisons based on characteristics derived from the analysis of all 3 independent leads modelling the chest belt recordings.

Supplementary Figure 6 shows the receiver operator characteristics for the prediction of all-cause mortality during study follow-up (panels A, B, and C) and for the prediction of appropriate ICD shocks (panel D). In all panels, the characteristic is shown together with its empirical 95% dual-sided confidence band. Area under the characteristic curve (and the corresponding 95% confidence interval) are shown in each panel. Panel A shows the characteristic derived from QRS loop non-planarity values while panels B and C show multivariable characteristics derived from a combination of the QRS loop non-planarity with heart rate and QRS-T angle values (options 2 out of 3 positive and 3 out of 3 positive are shown in panels B and C, respectively). These panels show that in terms of the receiver operator characteristics, the QRS loop non-planarity adds meaningfully to other ECG-based risk factors. The characteristic for the prediction of appropriate ICD shocks shown in Panel D was derived from the T wave loop non-planarity. The T wave loop non-planarity was the only ECG-based factor that led to a receiver operator characteristic with the area under the characteristic being significantly larger than 0.5.

The Harrel's concordance index C values (and their 95% confidence intervals) for all-cause mortality prediction by QRS-loop non-planarity, all-cause mortality prediction by T-wave-loop non-planarity, first appropriate ICD shock prediction by QRS-loop non-planarity, and first appropriate ICD shock prediction by QRS-loop non-planarity were 0.575 (0.542 - 0.605), 0.526 (0.493 - 0.560), 0.554 (0.512 - 0.595), and 0.568 (0.528 - 0.608), respectively.

Supplementary Figure 7 shows the incidence of all-cause mortality (red bars in panels A and C) and of first appropriate ICD shocks (blue bars in panels B and D) in populations quintiles sorted by QRS-loop non-planarity values (top panels A and B) and by T-wave-loop non-planarity values (bottom panels C and D). Chi-square p-values of the tests of distribution uniformity are shown in each panel. The differences from uniform distribution across the quintiles were all statistically significant apart

from the distribution of all-cause mortality in quintiles according to the T-wave-loop non-planarity values (Panel C).

Supplementary Figure 8 shows the Kaplan-Meier comparisons of follow-up all-cause mortality and of appropriate ICD shocks between patients with CRT and non-CRT defibrillators (panels A and B), and of the mortality differences between patients with QRS loop non-planarity below and above population median separately in patients with non-CRT defibrillators (panel C) and in patients with CRT-defibrillators (panel D). The number of patients at risk in these groups are shown below the panels in colours corresponding to the individual graphs. While the probability of all-cause mortality was significantly increased in patients with CRT defibrillators (panel A), there was only a borderline trend towards fewer appropriate ICD shocks among patients with CRT defibrillators (panel B). Panels C and D show that the mortality risk prediction by QRS loop non-planarity was maintained irrespective of whether the patients received CRT or non-CRT defibrillators. Since the CRT/non-CRT defibrillator type had only a non-significant influence on the probability of first appropriate ICD shocks, it was not surprising that the probability of the appropriate shocks was equally stratified by T wave loop non-planarity in both subgroups of patients who received CRT or non-CRT defibrillators (details not shown).

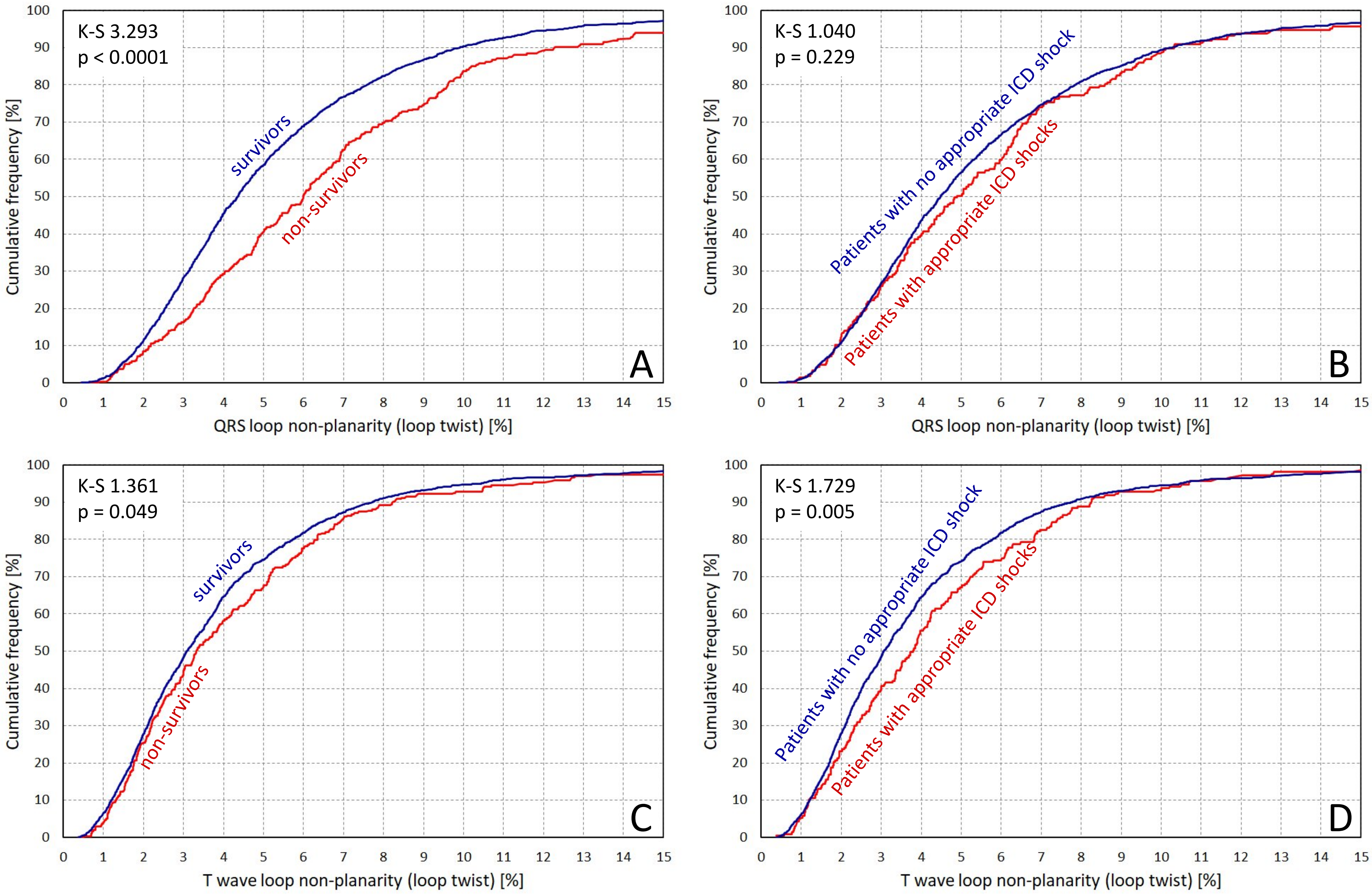

Supplementary Figure 3

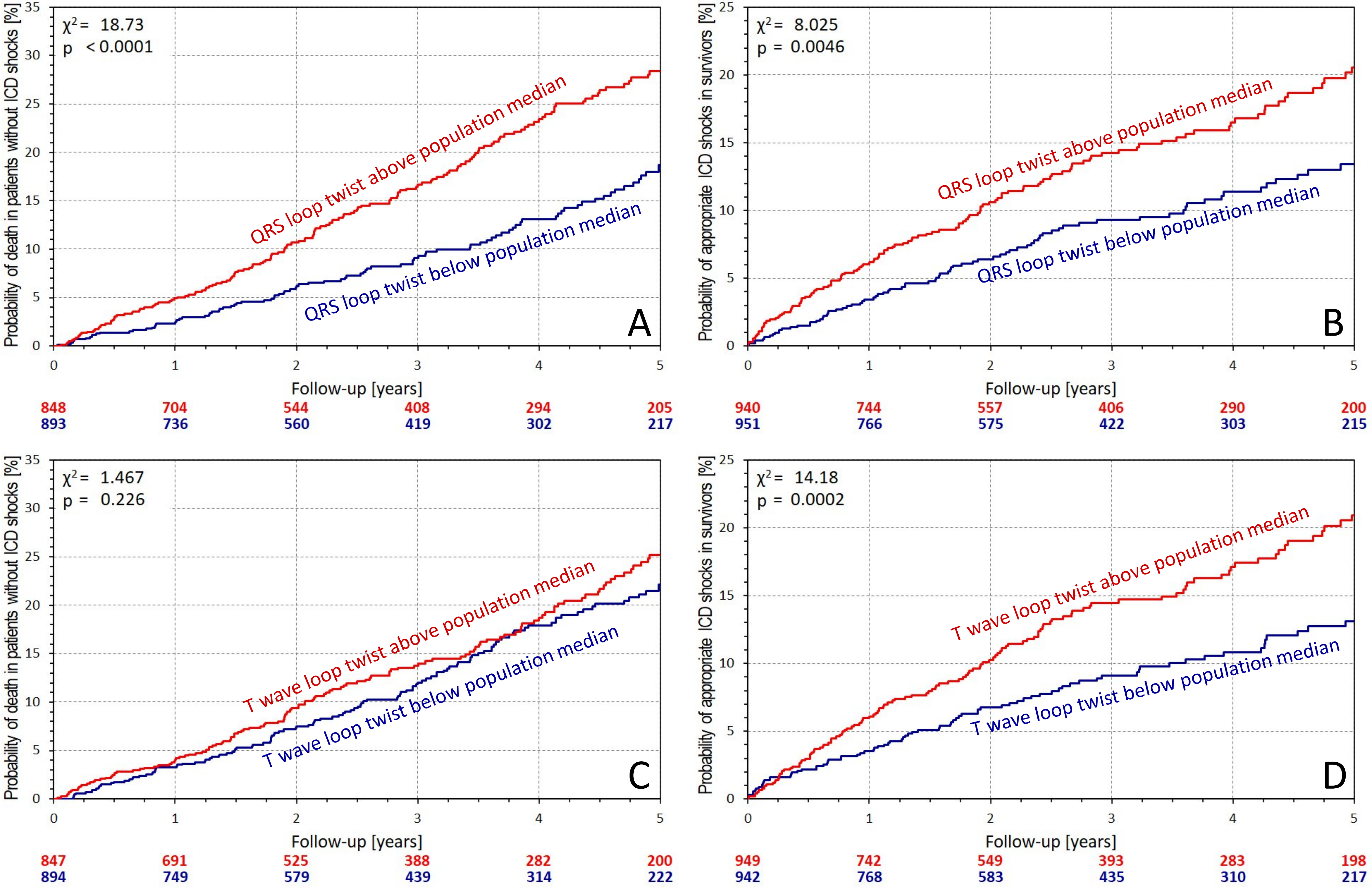

Supplementary Figure 4

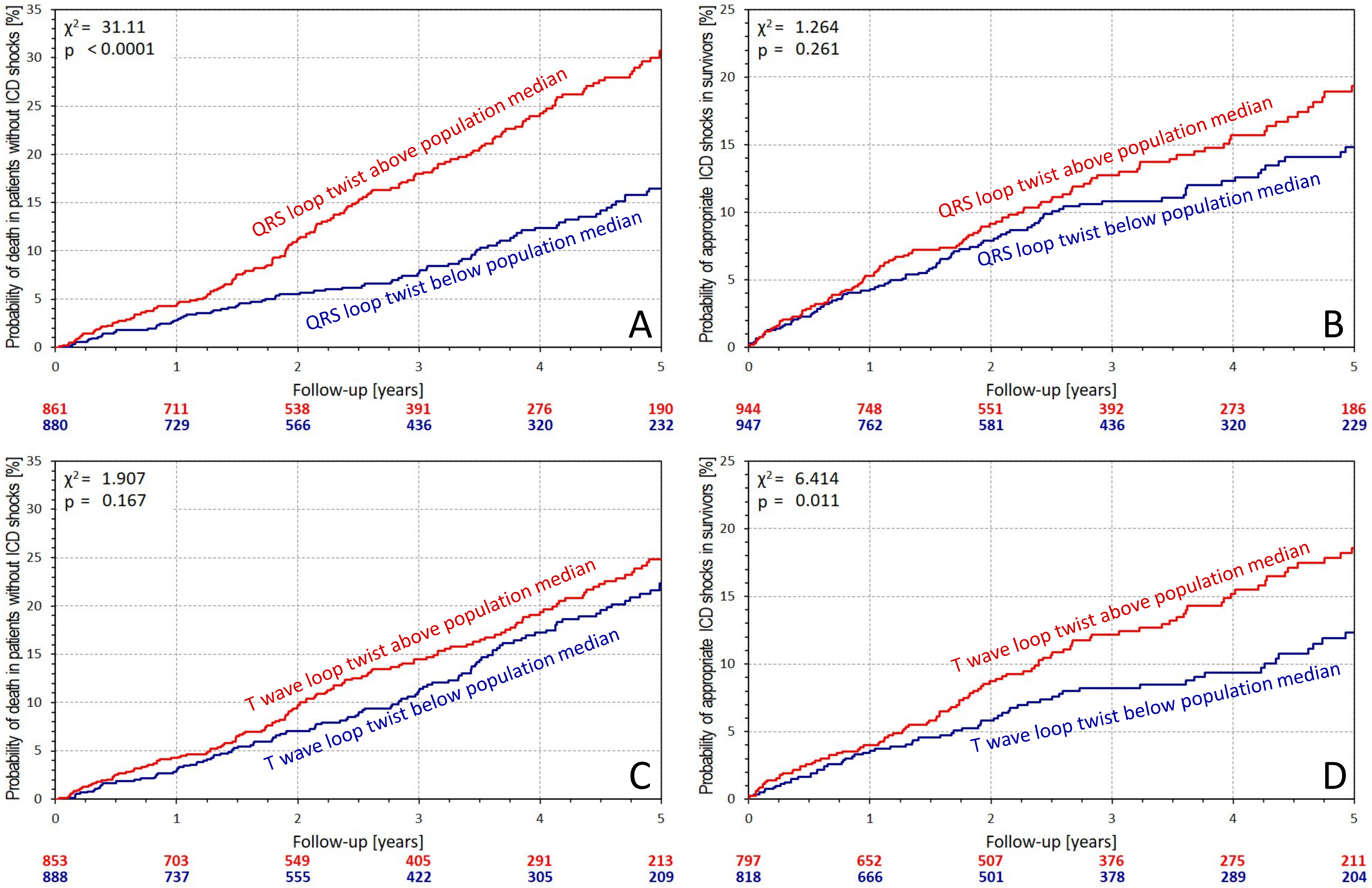

Supplementary Figure 5

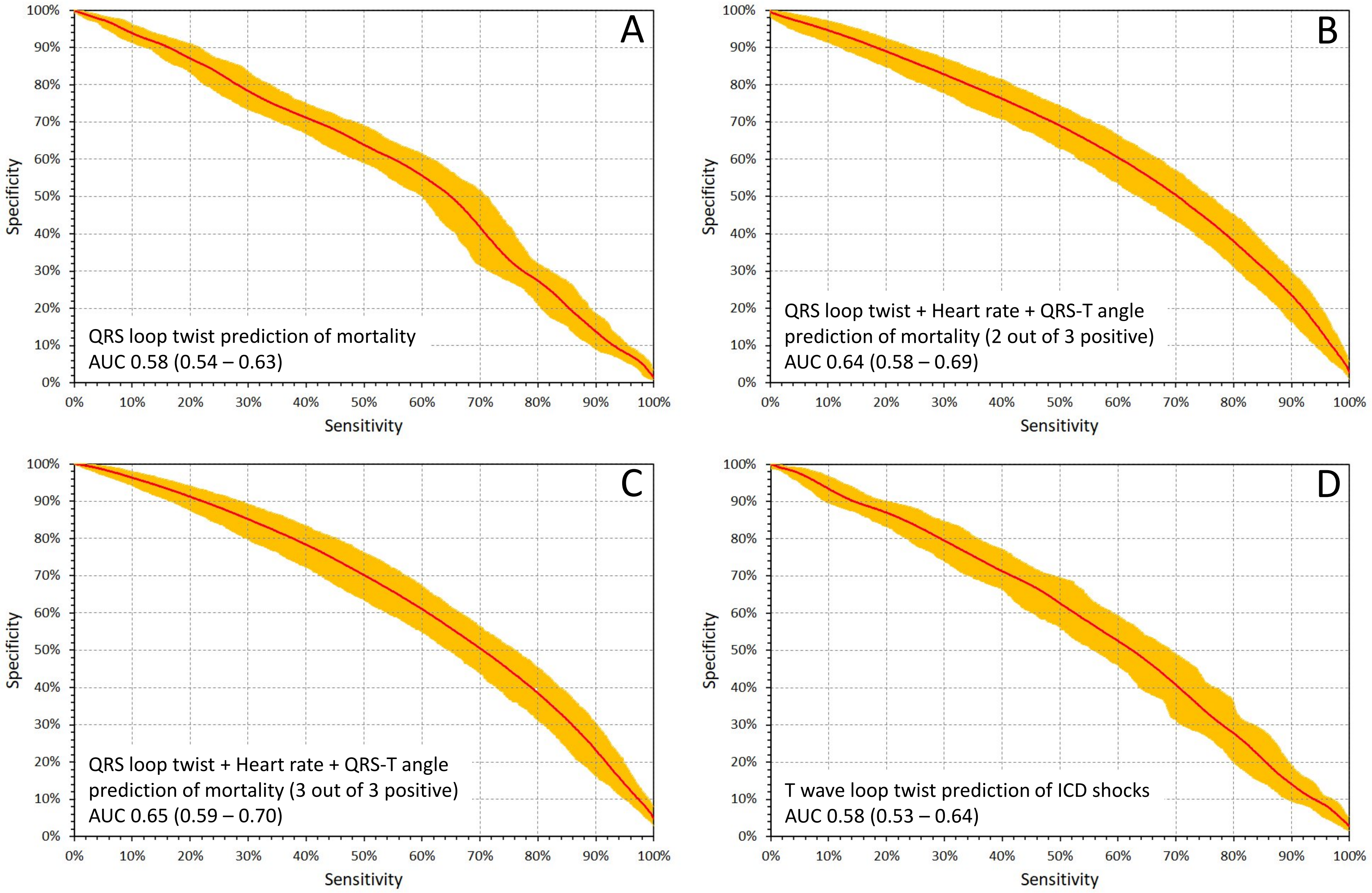

Supplementary Figure 6

BMJ Publishing Group Limited (BMJ) disclaims all liability and responsibility arising from any reliance placed on this supplemental material which has been supplied by the author(s)

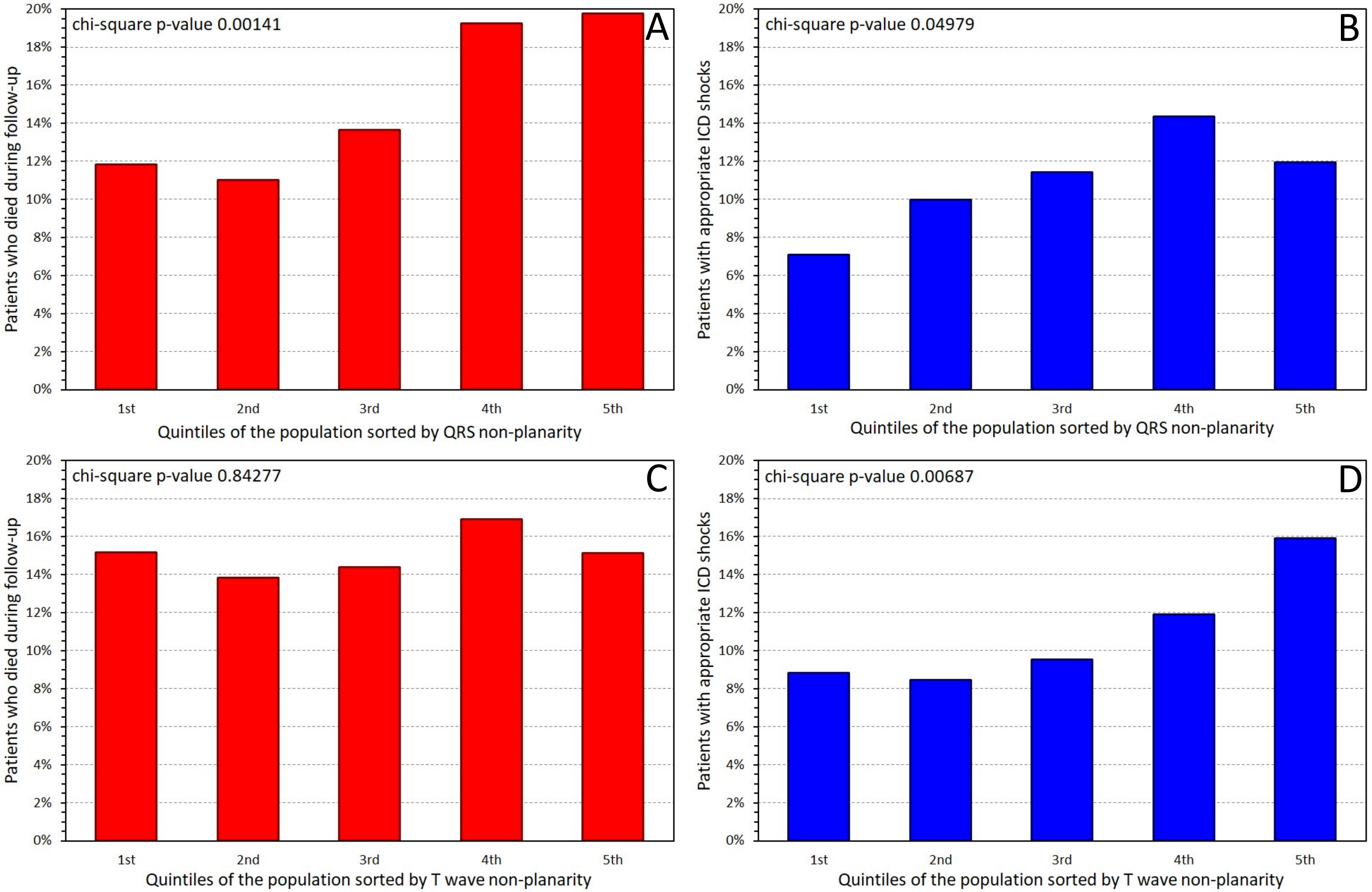

Supplementary Figure 7

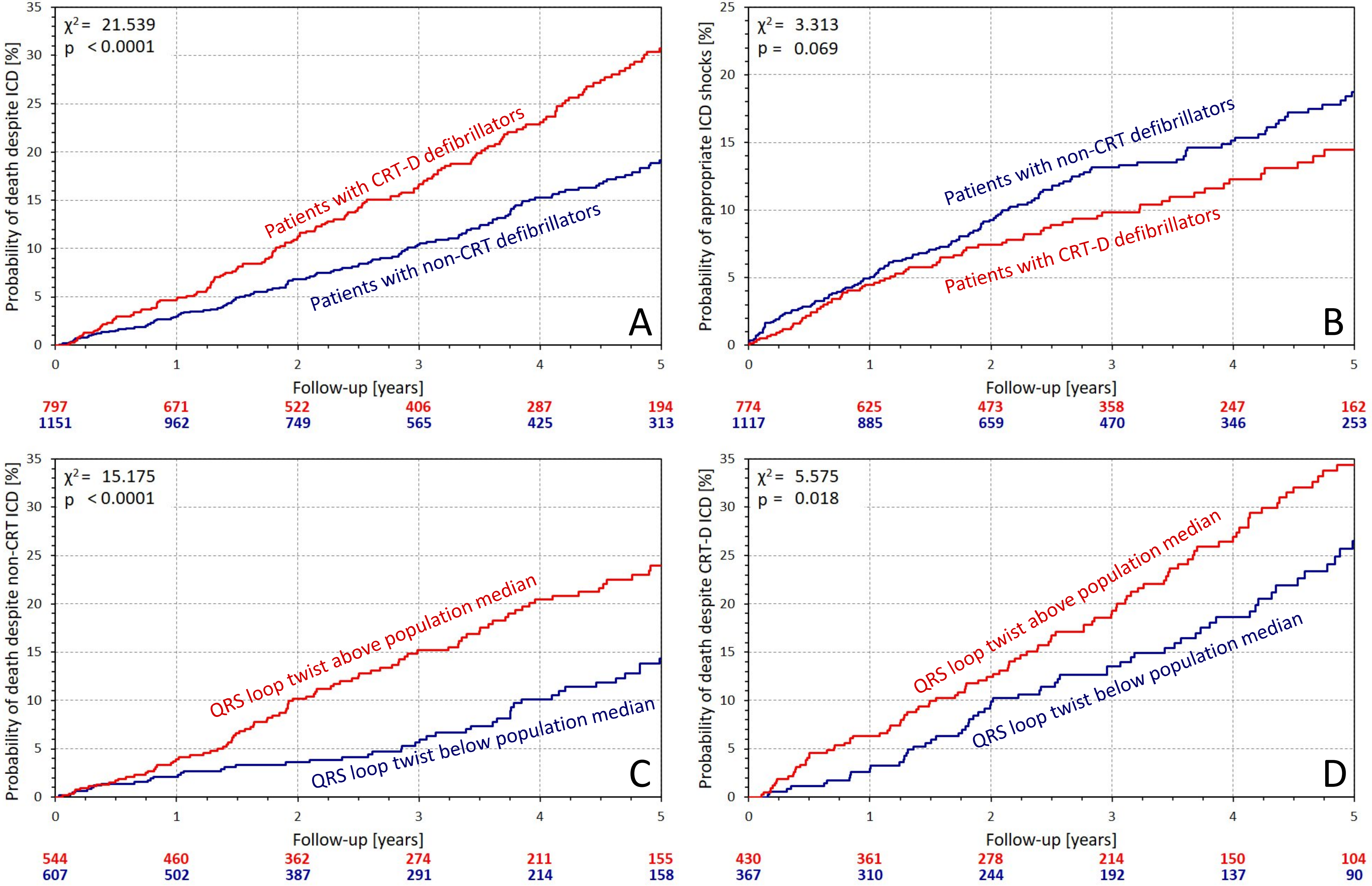

Supplementary Figure 8
