# Supplementary figures and images for: QRS complex and T wave planarity for the efficacy prediction of automatic implantable defibrillators
Source: Heart. 2023 Sep 15;110(3):178–87. doi: 10.1136/heartjnl-2023-322878 (PMC10850677; doi:10.1136/heartjnl-2023-322878)

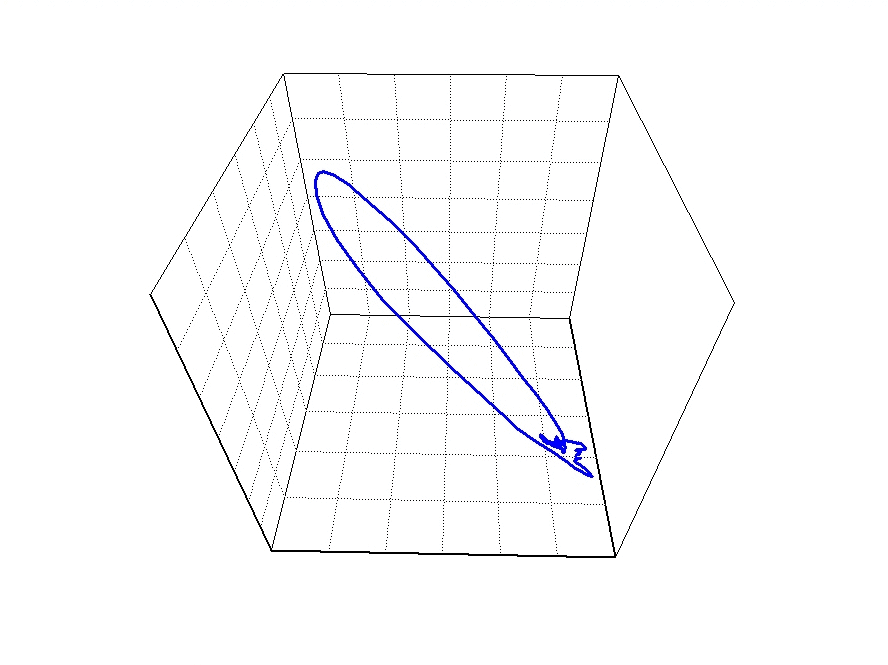

Supplement: Supplementary data [file heartjnl-2023-322878supp002.gif]

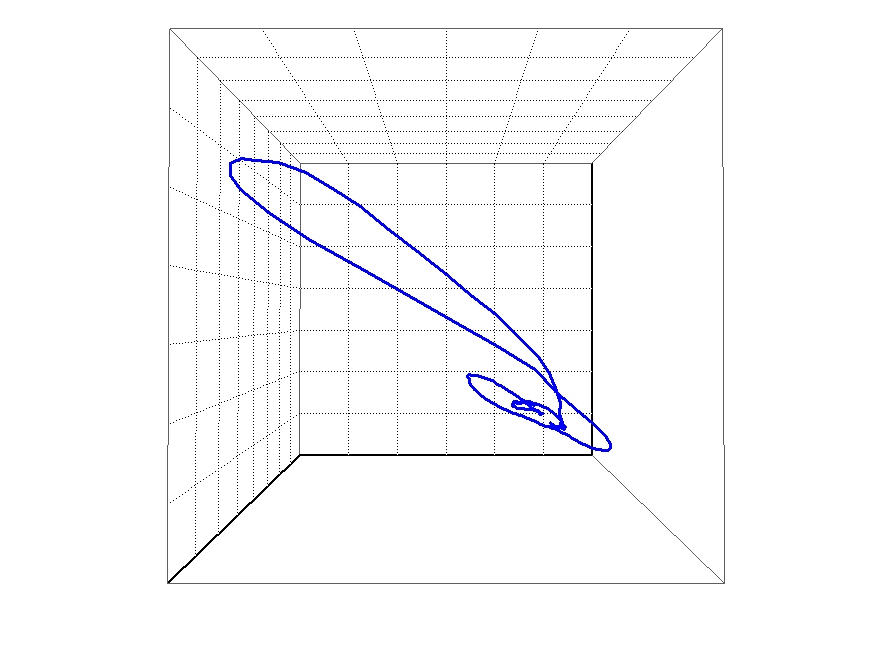

Supplement: Supplementary data [file heartjnl-2023-322878supp003.gif]
